# Sacral neuromodulation and peripheral nerve stimulation in patients with anal incontinence: an overview of techniques, complications and troubleshooting

**DOI:** 10.1093/gastro/gou015

**Published:** 2014-04-10

**Authors:** Andrew P. Zbar

**Affiliations:** ^1^ Department of Surgery and Transplantation, Chaim Sheba Medical Center, Ramat Gan, Israel and ^2^Assia Medical Colorectal Group Assuta Private Hospital, Tel Aviv, Israel

**Keywords:** anal incontinence, sacral neuromodulation, peripheral nerve stimulation, posterior tibial nerve stimulation

## Abstract

Sacral neuromodulation (SNM) therapy has revolutionized the management of many forms of anal incontinence, with an expanded use and a medium-term efficacy of 75% overall. This review discusses the technique of SNM therapy, along with its complications and troubleshooting and a discussion of the early data pertaining to peripheral posterior tibial nerve stimulation in incontinent patients. Future work needs to define the predictive factors for neurostimulatory success, along with the likely mechanisms of action of their therapeutic action.

## INTRODUCTION

Sacral neuromodulation (SNM or SNS) and, to a lesser extent, peripheral nerve stimulation (PNS) have effectively revolutionized the management of some intractable cases of anal incontinence (AI) and those unresponsive to or failing other more complex procedures. Because of its cost, it is still in somewhat limited (or restricted) use. In broad terms, SNM was approved for AI use by the U.S. Food and Drug Administration (FDA) on March 14^th^ 2011 and was supported by clinical practice guidelines in the United Kingdom in November 2004, where it has shown an equal benefit in mild, moderate and severe forms of incontinence. It was originally suggested as a technique for electrical stimulation of the bladder by Boyd in 1954, with the use of electrical pacing of the detrusor muscle in 1970, the utilization by Hopkinson of intra-anal pacing electrodes in 1972 and the use of the first formal SNM by Tanagho in 1982. Its use has been extended to those patients with double incontinence, as well as to patients with low anterior resection syndrome after low rectal and anal anastomosis, some patients with severe AI and associated rectal prolapse and those with external anal sphincter (EAS) defects—although its use for the latter condition is still controversial, particularly where the defects are considered large. Although long-term data are awaited, particularly in specialized groups, there has been an overall 75% efficacy with low morbidity and explantation rates and with very few contra-indications (including neural inaccessibility, prior implants, the regular need for MR imaging and active post-sacral sepsis).

Its clinical use in a patient cohort was first reported in *The Lancet* by Matzel *et al.* in 1995 [1]. It has (where available and affordable) become an accepted first-line treatment for patients who have failed to benefit from medical and behavioral therapies [2, 3]. The translation and development of techniques has occurred because of the excellent functional results obtained in urology, particularly for refractory urge incontinence, chronic urinary retention (Fowler’s syndrome) and detrusor hyper-reflexia. The period of neurostimulation in urology has been attributed to Tanagho, who first showed that sacral (S3) stimulation induced detrusor and sphincter function [4], with Schmidt outlining the basic set-up and technique of electrode placement [5]. Relatively recently, the introduction of a tined (barbed) lead has made a dramatic change in the surgical approach, with Spinelli *et al.* reporting that the success rate of this technique in selected patients for the permanent implant was significantly improved over a two-step technique initially using a temporary lead placement [6]. This has permitted a longer test period with the permanent lead before proceeding with the formal neurostimulator (IPG) implant.

## SACRAL NEUROMODULATION: TECHNIQUE AND OUTCOMES

Table 1 shows some of the larger international studies assessing the outcomes after SNM for AI and their complications [7–28]. In one systematic analysis by Maeda *et al.* in 2011, where an optimal outcome was obtained in 87% of 1159 pooled patients, the commonest cause reported for a suboptimal response included problems relating to the implanted lead [29]. The treatment in the correctly selected cases appears highly successful and the number of surgical re-interventions in this selected cohort is low (e.g. re-siting of the stimulator for pain, necessary exchange of the stimulator because of battery depletion, and the like). Sacral nerve stimulation has achieved an increasing worldwide application, where it is deemed suitable for many cases of passive and urge AI as well as in those cases both with and without a disrupted anal sphincter ring. More data are becoming available to expand its use in those patients normally destined for sphincteroplasty where there is a definitive EAS defect [30, 31], as well as in those cases with isolated IAS deficiency where an implant would normally be considered [32], in the incontinence and urgency associated with low anterior resection syndrome (with or without the construction of a neorectal reservoir) [33], and in those with partial spinal cord injury [34, 35].

**Table 1.** Reported initial series of sacral neuromodulation for anal incontinence

Author [Reference]	Year	Number	Temp^a^	Success	Follow-up (months)
Mellgren [26]	2011	133	n/a	90%	37
Uludag [27]	2011	50	n/a	84%	84
Boyle [28]	2011	50	37 (74)	54%	17
Wexner [23]	2010	133	120 (90)	83%	12
Michelsen [24]	2010	167	132 (79)	72%	24
Vallet [25]	2010	45	32 (71)	72%	44
Altomare [22]	2009	94	60 (64)	61%	74
Roman [18]	2008	18	18 (100)	78%	3
Vitton [19]	2008	5	5 (100)	100%	14
Tjandra [20]	2008	60	54 (90)	72%	12
Muñoz-Duyos [21]	2008	43	29 (67)	86%	35
Gstaltner [16]	2008	11	5 (45)	100%	not stated
Dudding [17]	2008	70	61 (87)	80%	24
Holzer [12]	2007	36	29 (81)	97%	35
Hetzer [13]	2007	44	37 (84)	92%	13
Melenhorst [15]	2007	134	100 (75)	79%	26
Gourcerol [14]	2007	61	35 (57)	61%	12
Faucheron [11]	2006	40	29 (73)	83%	6
Leroi [9]	2005	n/a	40	83%	6
Conaghan [10]	2005	5	3 (60)	100%	not stated
Rasmussen [8]	2004	45	37 (82)	86%	6
Ripetti [7]	2002	21	4 (19)	100%	15

^a^Success rate during temporary stimulation period (where recorded).

n/a = not available.

Although anorectal physiological testing and endo-anal ultrasound are part of the normal work-up, there is no evidence that these investigations predict for either outcome or response to temporary stimulation. In patients with an intact internal sphincter, an increase in mean resting pressure has been demonstrated with chronic sacral nerve stimulation [36], although the data are somewhat contradictory on this point [37]. This has been accompanied by an increase in maximal squeeze pressure in some series but not in others [38, 39]: an effect perhaps reflecting the variability of stimulation protocols. Although there is some debate suggesting that the sacral stimulation needs to be at least at the level of the sensory threshold (e.g. when the patient feels the stimulation somewhere in the perineum or vagina as a buzzing sensation that is tolerable and not disturbing) [40], satisfactory results have also been achieved when the stimulation level is sub-threshold. This latter point suggests that direct effects on the sphincter are less important for neurostimulatory success.

One of the contra-indications for this procedure is the patient with a complete spinal cord injury, where a lower motor neurone interruption of the reflex arc occurs or with a complete upper motor neurone lesion, where there is the risk of autonomic dysreflexia [41, 42]. There is, however, some debate concerning its value in those with associated congenital spinal abnormalities, such as *spina bifida* or complete sacral agenesis, where pre-operative radiology will show patency and accessibility of the vertebral foramina. Other contra-indications include patients with a prior history of epidural or spinal sepsis, neutropenia or other forms of immunodeficiency. In those patients with lumbar spinal fixation, antero-posterior and lateral pelvic radiographs should be performed to outline new bone formation, around which electrode placement may be precluded. It would appear that the use of sacral neuromodulation, when indicated, should not have an age restriction [43]. The results of sacral neuromodulation appear to show durability over time [26, 27] with an improvement in most of the AI-related quality-of-life parameters [23].

### The technique of SNM implantation and line insertion:

An X-ray of the sacrum should be performed if insertion of the electrode is predicted to be difficult. Otherwise there are no special investigations required. It has been suggested that external anal sphincter electromyography (EMG) is a significant positive predictive factor for a successful outcome [44], but in the study by Altomare’s group from Bari, Italy, the sensitivity of the test was poor and over half of the patients with an abnormal EMG still obtained benefit from the therapy. To date, no other predictive factors have been identified regarding who will benefit most from sacral nerve stimulation [45]. Patients who meet the screening criteria should not be refused testing based upon age, body mass index or length and severity of symptoms. Assessment of the integrity of the neural pathways is not routinely performed. It should be remembered that InterStim (Medtronic Minneapolis, MN) therapy has the potential to interfere with co-existing stimulator implants, most notably cardiac pacemakers, although the two may be compatible for implantation in most patients. In this circumstance, it is suggested that a low pulse width with a bipolar stimulation at a frequency >30 Hz be used for SNM work, since low frequencies are more likely to inhibit the cardiac pacemaker [46]. Guidelines also suggest routine cessation of anticoagulant therapy and covering patients with peri-operative heparin following standard protocols.

The technique for general or local anesthesia is case-based. The author favors use of general anesthesia in selected cases and in children, but local anesthesia in some patients after discussion, particularly in those with AI after congenital anorectal anomaly reconstruction, where it may be advantageous to use the sensory response as a better guide to intra-operative efficacy, or where lead insertion might prove difficult. With acute stimulation, a ‘buzzing’ sensation can be felt anywhere from the coccyx to the clitoris, but more commonly in the vaginal *introitus* or the perineal body, where the sensation is dependent upon the amplitude of stimulation, the frequency and the pulse width of stimulation and the depth and proximity of the electrode to the relevant nerve root. Poor sensation in patients should be coupled with the typical motor response of the anal ‘bellows’ where the anus will balloon and pucker during stimulation. For these purposes, the anesthetist needs to know that the procedure is performed prone (where a protected airway is required) and that the use of long-acting muscle relaxants is contra-indicated. If the patient is intubated, this should be performed without muscle relaxation or by using a very short-acting opioid analgesic.

Antibiotic prophylaxis is traditionally used, although infection rates are small but, given the temporary period of stimulation, it would seem mandatory. It is of interest to note that half of the temporary leads will show bacterial colonization at the time of their removal [47]. Fortunately pacemaker pocket sepsis is rare at <2% overall, with a slightly higher rate in staged cases. It is suggested that, as meta-analyses have reported a reduced risk of serious infections with cardiac pacemaker implantation, this should be translated to SNM without specific recommendation for a particular antibiotic of choice. The author uses a single intra-operative dose of a broad-spectrum cephalosporin.

There are numerous ways of finding the S3 sacral foramen using specific bony landmarks. Firstly, the patient should have a pillow placed under the pelvis to remove any lumbar lordosis. If a horizontal line is drawn parallel to the top of the greater sciatic notch (which is also the site of the sacral promontory), this is then marked by a pen. The sacral foramen lies one finger’s width off the center at this level. Another way is to locate the greater sciatic notch on both sides, draw a horizontal line between them, and define the midline so that the lines cross. The S3 foramen will lie under a point one finger’s width laterally to the cross and one finger’s width up from this point. In Maastricht, Baeten’s group prefers to mark a point halfway between the top of the sacrum and the tip of the coccyx, and then to mark the S3 foramen off the midline as before from this point. The author uses a combination of these techniques as verification.

A needle is passed through the S3 sacral foramen by approaching it at a 60° angle to the skin, hitting the sacral periosteum and then advancing into the foramen. The acute in-theater testing is usually performed with a pulse width of 210 μs and a frequency of 14 Hz. Perineal, vaginal or penile stimulation may be experienced with overly deep placement of the lead at the S4 level, whereas a sensation of stimulation in the buttock or the leg is probably indicative of an S2 root stimulation. Equally, the motor response relates to the level of stimulation, whereby the bellows response will be accompanied by flexion of the big toe or of the forefoot, whereas this will be absent with S4 stimulation. Flexion of the entire foot or internal rotation of the leg is indicative of a higher S2 location of the stimulating wire, which should be repositioned, as it will lead to undue pain. Once the S3 root is located, the depth and the angulation may be slightly changed so that the lowest stimulation voltage can be used. The author does not use multiple leads or bilateral SNM in practice unless there is a very poor response at high stimulation amplitudes, and does not use the temporary wire (Medtronic Minneapolis, MN model 3057) as it requires an extension cable, which is cumbersome.

The permanent, tined lead is nowadays connected to the InterStim II (Medtronic Minneapolis, MN model 3058) as there is no need for an extension lead (which could break between stages) although this results in shorter battery life. The InterStim I (Medtronic model 3023) has now become redundant.

A rigid dilator within an introducer sheath is then passed over the guide wire, under radiological guidance, to a depth such that the distal tip of the sheath lies at the anterior cortex of the sacrum on screening imaging. There are two types of permanent lead (Medtronic model 3889 and model 3093), each with a four-point electrode system for stimulation, but with variations in spacing of the electrodes. The stimulation should ideally obtain a sensory or a motor response (or both) for a low amplitude stimulation of all of the electrode points but, if this cannot be achieved, the responding electrode can be best positioned so that a field is created with the next electrode. This will provide the greatest chance of switching post-operative programming. Fluoroscopically, this should appear so that the most proximal electrode (electrode number 3) lies within the sacral foramen, electrode 2 is level with the anterior sacral cortex on sagittal imaging, and the two most distal electrodes (electrodes 1 and 0) lie fully anterior to the sacrum. White markers on the system are the points at which the electrodes are exposed through the sheath. As withdrawal of the sheath begins, there is a ‘give’ sensation as it passes the first tine and, following this, further repositioning of the lead is precluded. After lead positioning, a special trochar with a plastic introducer is inserted near the lead, being careful not to damage the lead by passing the tunneller from the lead side and passed to the contralateral side. The temporary lead should be secured with adhesive and povidone iodine ointment-covered transparent dressings, which can be observed and which are water resistant. This permits showering after unplugging of the external stimulator. Swimming and bathing are not permitted.

For the pacemaker implantation stage, the wire is simply disconnected and re-attached to the InterStim II with the supplied, special screwdriver. A subcutaneous pocket for the implant is then made deep into Scarpa’s fascia on the ipsilateral upper buttock, distant from the iliac crest. The implant should lie superior to the lead insertion point for further radiography and the implant can be sutured in place at two points to prevent migration or rotation. In the first stage, the lead can be placed deeply, so that it is less likely to be damaged during dissection of the second-stage procedure. Equally, the lead should be placed behind the stimulator so that further dissections are less likely to damage the lead. It has been suggested that telemetry is better when the writing on the implant is positioned uppermost and where the IPG can be used as an anode in a unipolar configuration.

No consistent physiological changes have been demonstrated with SNM. Assessment of success is therefore based on the patient’s subjective measurement of the outcome. Published studies have used diary card assessment or continence scores to assess the outcome of temporary stimulation, which is considered successful as a test if there is at least a 50% reduction in the number of incontinence episodes over a given period, or a similar increase in incontinence-free days. This sort of assessment is crude, however, if a patient is severely incontinent and may not necessarily alter his or her habit of using pads. The key metric would more usefully be an improvement in daily activity, meaning that incontinence no longer drives (or restricts) the structure of the patient’s average day.

In summary, the technique of SNM has become fairly standardized over the last five or more years, and has moved from positioning of a temporary lead to deployment of the permanent lead, but remains a two-stage process of initial temporary stimulation pending a decision regarding permanent second-stage pacemaker implantation. The procedure is usually performed unilaterally but, if difficulty is encountered, a bilateral approach may be used. In-theater stimulation of the quadripolar lead (model 3093) is not standardized but should take the form of external stimulator settings around a pulse width of 210 μs, rate of 14 Hz and an amplitude of 10 V for all four positions (0, 1, 2 and 3) of the lead responding for response. Prospective randomized data show that the two-stage implant technique of SNM has a higher success rate when compared with the single-stage method, despite a prior positive PNE response, and effectively reduces the re-operation rate and overall procedural cost [48–50].

### Complications of SNM

Complications may broadly be divided into test-stimulation-related and implantation-related problems. Most relate to lead migration (about 12%), with pain (3%) and infection (10%), with reoperation (15%) for a combination of events including attenuated response, infection, IPG site pain and lead migration. It has been suggested that poor responses require formal impedance measurement, although this is not standard practice [51]. Lead migration is usually resolved by reprogramming and usually does not require a new lead to be inserted. In some cases there is an accommodation to stimulation, which does not respond to an increase in stimulation amplitude, and this may ultimately require a repeat insertion or a contralateral lead insertion.

Problems relating to response may also occur as a result of impedance resistance—with attenuation of electron flow through the circuit—such that current does not flow if there is excessive resistance. Impedance describes the resistance to the flow of electrons through a circuit. The reverse—where there is very little resistance—will shorten the battery life. The set-up is such that the current will travel from the electrode, through the patient, to a separate electrode (a bipolar system) or through the patient to the IPG pacemaker (neurostimulator-unipoloar system). Impedance measurement can act as a troubleshooting technique, checking the system’s integrity in patients who lose SNM efficacy. In this setting, high resistance levels (>4KΩ) indicate an open circuit, which is usually due to a fractured lead, loose connections or both. This can also define the problem at each electrode and is handled by reprogramming to move away from the broken electrode. Infection can be mild or severe with the latter requiring explantation of the IPG or, short of this, the first option of debridement of the wound, antibiotics and a delayed secondary IPG implantation. Pain at the IPG site may, in many cases, also be managed by reprogramming. Some patients experience pain from monopolar stimulation, which may be negated by changing the stimulation to a bipolar mode. Reprogramming will also deal with most cases where pain may be due to leakage of current, by changing the mapping or the pulse width and its rate. Most patients, when reassured, will live with these minor forms of irritation.

### SNM troubleshooting

In SNM troubleshooting, there needs to be agreement over the terminology of treatment failure [52]. Suboptimal outcomes show up as a lack of clinical efficacy or a loss of efficacy after initial success. This is usually a result of a suboptimal location of the permanent lead or excessive perilead fibrosis over time. Of course, mechanical factors can occur with electrode migration, broken leads, dislodged and loose connections, as well as progression of the actual cause of AI, particularly if there is an underlying neurological condition.

Pain is also a common complaint (in about 15% of cases) and may be due to local factors as well as stimulation-related. The IPG may have associated hematoma, cellulitis, local allergic reaction or erosion. Most infections will tend to occur early and be secondary to s*taphylococcal spp* [53]*.* Many patients may also report adverse function secondary to unwanted periods of stimulation, particularly during sleep. The impact of SNM on sexual function and during pregnancy is poorly understood, since some patients may find stimulation unpleasant during periods of heightened sexual drive [54, 55]. Overall, data would suggest that, when SNM is used for functional bowel disease, about half of the patients will experience at least one device- or treatment-related adverse event (although often of a minor nature) with a surgical revision rate that is less than 20% overall. Surgical revision is mostly related to pain around the IPG or infection, with a low explantation rate of 5–10%.

**Figure 1.**
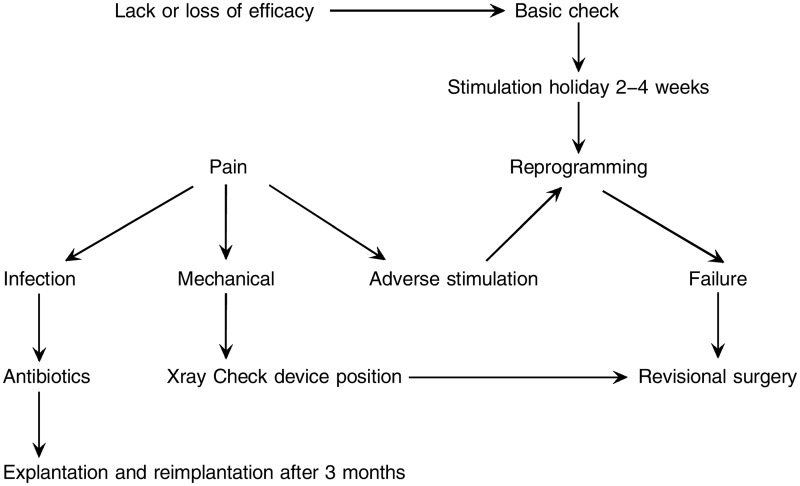
Algorithm for troubleshooting sacral neuromodulation (Patterned after Matzel KE, Maeda Y. *The problematic sacral neuromodulation.* In: *Reconstructive Surgery of the Rectum, Anus and Perineum.* AP Zbar, RD Madoff, SD Wexner (Eds). London: Springer, 2013).

It is useful to have an established algorithm for SNM troubleshooting, which has a co-ordinated approach to lack or loss of SNM efficacy, to pain and infection or to adverse stimulation, as shown in Figure 1.

The first step is a basic check to determine whether the system is operating normally. This also assesses the patient’s familiarity with the system. A short period with the system turned off may allow a re-regulation and efficacy, often at a lower amplitude, although this may require off-periods of up to one month. Clinical examination will detect local IPG problems that may account for pain. Testing as stated above will evaluate the impedance of the system. An abnormal impedance (<50 Ω or >4 KΩ) may be associated with electrical system disruption (including lead breakage, disconnection, a loose connection or lead tension). High impedance (>4 KΩ)—an open circuit—indicates lead breakage, disconnection, or a loose connection, whereas low impedance is a short-circuit, which results from a tight connection or body fluid intrusion around the connection. Most analysis leads to slight reprogramming, since the easiest thing is to change the pole combination and amplitude. There is no standardization of reprogramming, although it should be performed relatively systematically [56]. The sensory effect of stimulation may be tested first in the unipolar mode (as four combinations) and then in the bipolar mode (as 12 combinations), to identify the best combination for the patient so that there is always a balance between pain and stimulation. Changing the electrode configuration, focusing the effect nearer the anus, will usually eliminate problems of increased bladder frequency or troublesome vaginal stimulation and is generally thought to be more efficacious in AI. As bladder frequency dominates at low frequencies, increasing the frequency may obviate this problem in some cases. It is not recommended to use high pulse widths (>450 μs) or high frequencies (>50 Hz) as this can theoretically lead to permanent neural damage.

Surgical revision may be used after reprogramming failure for lead migration, which is nowadays less common using the tined lead. Lead migration may be anterior or posterior and may be detected by a lateral sacral X-ray, which also defines lead breakage. Minor migration may be overcome by increasing stimulation amplitude. Failure of this change may result in the need for a new lead to be positioned, either ipsilaterally in a different sacral foramen or contralaterally [57, 58]. The overall battery life of the implant depends on the amplitude and the mode of stimulation; for example, if used within the normal parameters, the life of the InterStim II device should be of the order of seven years or so. The current role of bilateral stimulation in the failing case is unclear, particularly as the afferent outflow is variable from the pelvis, so there will be some cases where isolated S3 stimulation will be insufficient [59, 60]. Most reprogramming, although required during the functional life of SNM, is of a minor nature and does not appear to increase over time.

## PERIPHERAL NERVE STIMULATION AND ANAL INCONTINENCE

Posterior tibial nerve stimulation (PTNS) was first used for urinary incontinence in 1983, independently by Nakamura and McGuire *et al.* [61, 62] and translated for fecal incontinence by Ahmed Shafik in 2003 [63]. This latter technique was modified by Queralto *et al.* in 2006 to a simpler transcutaneous method [64], as an extension of the transcutaneous electrical nerve stimulation (TENS) technique for pain, where a series of small open-label studies has been reported (Table 2). The technique takes advantage of spinal and supraspinal neuromodulation (as discussed), during and after stimulation, applied to the posterior tibial nerve, which contains sensory, motor and autonomic component fibers derived from the 4th to the 5th lumbar- and the 1st to the 3rd sacral roots [65]. There have been some recent seminal papers on the technique and the preliminary results [66–70].

**Table 2.** Early reported results of peripheral nerve stimulation therapy for patients with anal incontinence

Author [Reference]	Year	Number	Results	Follow-up (months)
Shafik [63]	2003	32	84% improved	30
Queralto [64]	2006	10	80%	3
Mentes [66]	2007	2	30% improved	6
Vitton [72]	2009	12	40%	not stated
de la Portilla [67]	2009	16	40%	6
Govaert [73]	2010	22	50%	12
Boyle [74]	2010	31	65%	3
Findlay [69]	2011	13	65%	1

As with SNM, PNS has variably reported treatment protocols with differences in duration and electrical frequency, with portable neuromodulatory pulse generators delivering current up to 9 mA directly to the posterior tibial nerve. The nerve is accessed immediately above the medial *malleolus* with either a needle or an adhesive electrode, supplemented by a grounded adhesive electrode [71]. Correct placement of the electrode is determined by the induction of plantar flexion or abduction, where PNS is induced at the highest available current that does not result in a motor response. The technique may be supported by patient modification as a ‘top-up’ to medical sessions of stimulation [72]. The role of the technique is uncertain, although clearly it is cheaper than SNM, with very few contra-indications (coagulopathy and local neuropathy) and with inherently few complications.

In the original work by Shafik [63], 32 patients with intact sphincters received temporary stimulation by a needle electrode for short periods of time, with about a 50% overall improvement, although there was a 30% relapse rate. In the study by Queralto *et al.* [64], ten cases with idiopathic AI were treated similarly, with improvement in eight patients. In a small study from Turkey by Mentes, patients with dual incontinence after partial spinal cord injury were treated, with the first reported improvements in quality-of-life parameters [66]. In a longer-term study from Spain by de la Portilla, in 10 patients with a heterogeneous group of AI cases, there was a 44% improvement with coincident manometric improvement in some cases, where the benefit was experienced over a medium term of follow-up [67]. In the first multicenter study by Govaert *et al.* conducted from the Netherlands, Spain and Italy, a mixed population of 22 AI cases, including some patients with clear EAS defects, showed improvement in 14 cases, in which most responders maintained their improvements over one year [73].

The limited number of available studies—and their heterogeneity—leaves the role of PNS unclear, since the data is currently uncontrolled and studies have limited power. There are no studies comparing other treatment modalities with PNS. The role of PNS in those patients not responding to SNM or non-responsive to SNM also remains unclear and is an area for future study. It is also uncertain whether those poor prognostic factors in the use of PNS in urological disorders translate to those with AI [74]. The details of stimulation sessions and their frequency will also need to be determined because, in urology, more frequent treatments appear to offer better results. Multi-center studies are underway and currently recruiting in the Netherlands (Maastricht clinical trials. gov NCT00974909) and France (Rouen NCT00977652), which will make blind comparisons between PNS and placebo over a minimum of 12 months.

Potentially, PNS may finally fit into current treatment regimens by obviating the need for more invasive procedures with higher operative risk and cost, and could provide an interim control measure for those awaiting more definitive interventions, as well as releasing an effective means of patient-directed therapy which can be conducted independently at home. There is also the potential for patient-directed treatment. Where this treatment may sit in relation to conventional acupuncture is also uncertain and needs further development [75], as PNS tibial stimulation corresponds to the SP-6 acupuncture point (called the ‘sanyinjiao’ or ‘spleen-6’ position point). The use of PNS appears to show non-dominance, so it does not matter which leg is stimulated [76]. Although stimulation techniques are similar with SNM, it has been suggested that a shorter pulse width and a higher frequency is required for PNS treatment, although the optimal duration and frequency is unknown. In this respect, there is some evidence of a residual benefit after PNS cessation, as Govaert *et al.* have suggested that repeated top-up treatments can be reduced after initial success [73]. The cost of the treatment is not excessive, since a re-usable PTNS stimulator device costs £800 sterling, with a box of single-use percutaneous needle electrodes adding a further £380 whereas, in the UK, the equipment costs alone for SNM can approach £10 000 [77–79]. Further studies determining the role of transcutaneous (as opposed to percutaneous) strategies are required to fit into home-based, cost-effective regimens of therapy. By comparison, SNM will achieve complete continence in 41–75% of cases; however, adverse events can occur in about 10% or more, including device infection and lead migration, which necessitate revisional surgery in about 5% of patients. Future assessments of PNS in open-label studies will need to address these response and complication outcome data. Table 2 shows the preliminary reported outcomes using PNS in patients presenting with AI. Future studies will compare PNS with SNM and decide where PNS should fit within the AI algorithm. Delineation of its effect over placebo is required, along with comparisons with acupuncture treatment, assessing whether it induces changes in rectal sensory perception, striated muscle function and its effect on involuntary anal relaxations and rectal contractions.

In summary, in the future, many forms of AI will be treated primarily by neurostimulatory therapies. Comparative studies of SNM and PTNS are required to better define those cases suitable for each type of treatment, given their current costs and limited availability. Presently, there are no predictive clinical or physiological factors for success in treatment and further work needs to better define their purported mechanisms of action.

**Conflict of interest**: none declared.
